# PIRSitePredict for protein functional site prediction using position-specific rules

**DOI:** 10.1093/database/baz026

**Published:** 2019-02-26

**Authors:** Chuming Chen, Qinghua Wang, Hongzhan Huang, Cholanayakanahalli R Vinayaka, John S Garavelli, Cecilia N Arighi, Darren A Natale, Cathy H Wu

**Affiliations:** 1 Center for Bioinformatics and Computational Biology, University of Delaware, Newark, DE, USA; 2 Department of Computer and Information Sciences, University of Delaware, Newark, DE, USA; 3 Protein Information Resource, Georgetown University Medical Center, Washington, DC, USA

## Abstract

Methods focused on predicting ‘global’ annotations for proteins (such as molecular function, biological process and presence of domains or membership in a family) have reached a relatively mature stage. Methods to provide fine-grained ‘local’ annotation of functional sites (at the level of individual amino acid) are now coming to the forefront, especially in light of the rapid accumulation of genetic variant data. We have developed a computational method and workflow that predicts functional sites within proteins using position-specific conditional template annotation rules (namely PIR Site Rules or PIRSRs for short). Such rules are curated through review of known protein structural and other experimental data by structural biologists and are used to generate high-quality annotations for the UniProt Knowledgebase (UniProtKB) unreviewed section. To share the PIRSR functional site prediction method with the broader scientific community, we have streamlined our workflow and developed a stand-alone Java software package named PIRSitePredict. We demonstrate the use of PIRSitePredict for functional annotation of *de novo* assembled genome/transcriptome by annotating uncharacterized proteins from Trinity RNA-seq assembly of embryonic transcriptomes of the following three cartilaginous fishes: *Leucoraja erinacea* (Little Skate), *Scyliorhinus canicula* (Small-spotted Catshark) and *Callorhinchus milii* (Elephant Shark). On average about 1200 lines of annotations were predicted for each species.

## Introduction

Experimental characterization of protein function lags far behind the pace of high-throughput genomic sequencing; thus, the protein function characterization is heavily relying on computational methods. Many computational methods for protein function prediction have been developed in the past decades (1). Most such methods focus on ‘global’ annotation, such as molecular function, biological process and presence of domains or membership in a family (2–8). Only a few methods provide fine-grained ‘local’ annotation of functional sites based on protein structural data at the level of individual amino acid (9–11). The global and local methods are complementary. Site-based local method can inform the global method to be cautious about certain annotations if the related site feature is not present or differs from the one expected. For example, the protein entry Q73WF2 (https://www.uniprot.org/uniprot/Q73WF2) in UniprotKB is pyridoxal 5′-phosphate synthase subunit PdxT in *Mycobacterium paratuberculosis*. Glu-170 and Asp-172 are present instead of the conserved His and Glu, which are expected to be the active site residues, respectively.

The computational methods for functional site prediction can be broadly divided into following types (12) and various hybrids of these types (13): (i) methods based on genomic context; (ii) methods based on sequence; (iii) methods based on structure; (iv) methods based on literature and text mining; and (v) machine learning methods. For example, various PTM (Post-translational modification) site prediction approaches and online platforms have been previously well reviewed (14–17). Other than computational methods, there are resources focusing on experimentally determined sites, such as Phospho.ELM storing *in vivo* and *in vitro* phosphorylation data extracted from the scientific literature and phosphoproteomic analyses (18), and Mechanism and Catalytic Site Atlas for enzyme reaction mechanisms and active sites (19). PIR (Protein Information Resource) site rule (PIRSR) system makes use of structural-guided sequence alignments and profile HMMs (Hidden Markov Models), as well as taxonomic scope and literature evidence for template sequences. This hybrid approach enables multifaceted advantages than many traditional approaches, for example where many prediction efforts process only the putative central part of the recognition motif in their score function (20, 21).

As part of the UniProt Consortium, we contribute to the UniRule automatic annotation system with expert-created rules that annotate at the local level; these are known as site rules or PIRSRs. PIRSR relies on the PIRSF (PIR Super Family) family classification system (22), an expert-curated classification system that classifies protein sequences into families, whose members are both homologous (evolved from a common ancestor) and homeomorphic (sharing full-length sequence similarity and a common domain architecture). PIRSF is a member of the InterPro database (23) that includes ‘signatures’ representing protein domains, families, regions, repeats and motifs from major protein signature databases. The site rules are curated and defined by structural biologists on the basis of known structural and experimental data in characterized members of the corresponding PIRSF family. The rule determines the conditions under which a given annotation is applied (for example, a given annotation may only apply to a specific amino acid type). We have now opened our system to the community by developing PIRSitePredict, a stand-alone software package.

In the rest of the paper, we present PIRSitePredict that supports and maintains curation of position-specific rules and applies those rules to predict functional sites for protein sequences. We describe the methods and algorithms that provide annotation of functional sites using position-specific conditional template annotation rules. We show the utility of PIRSitePredict with the following two applications: UniProtKB automatic annotation and Genome/Transcriptome annotation.

## PIRSitePredict system

### Overview

**Figure 1:**
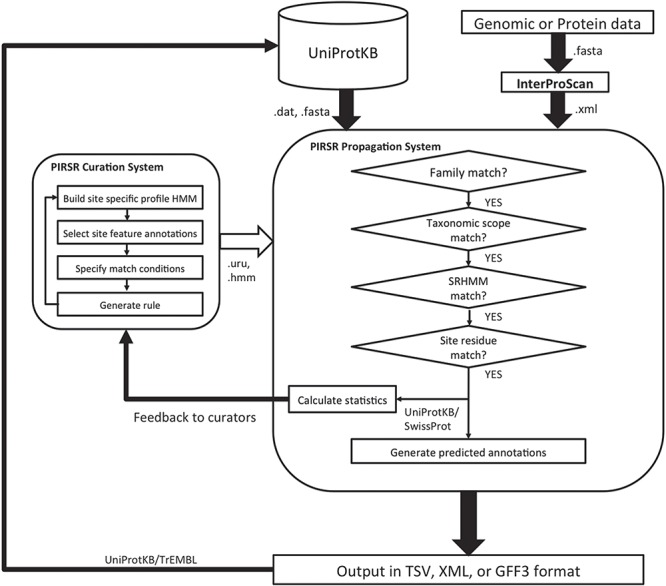
PIRSitePredict system overview.

The overall architecture of PIRSitePredict is shown in Figure 1. The core is the PIRSR system, which has the following two components: one that supports curation of rules (curation system), while the other applies those rules to predict functional sites in protein sequences (propagation system). To ensure our software is easy to use for our users, the system is designed to take the XML output from InterProScan, which is widely used by the annotation community. As PIRSitePredict is intended for fine-grained analysis of proteins within a single family, users should first run their protein or genomic sequences through InterProScan to ascertain the appropriate set of rules to test. PIRSitePredict uses InterProScan results and related organism information (Kingdom/Sub-taxon) as inputs, applies the curated PIRSRs, site-specific profile Hidden Markov Models (SRHMMs) and template protein sequences (a template protein is a representative protein in a protein family that has 3D structure with experimental evidence for the functional sites and modifications) to predict the functional sites for protein sequences matching InterPro signatures. The prediction results can be produced in the following three formats to facilitate interoperability: TSV (Tab-Separated Values), eXtensible Markup Language (XML) and Generic Feature Format (GFF3).

### PIRSR curation

**Figure 2:**
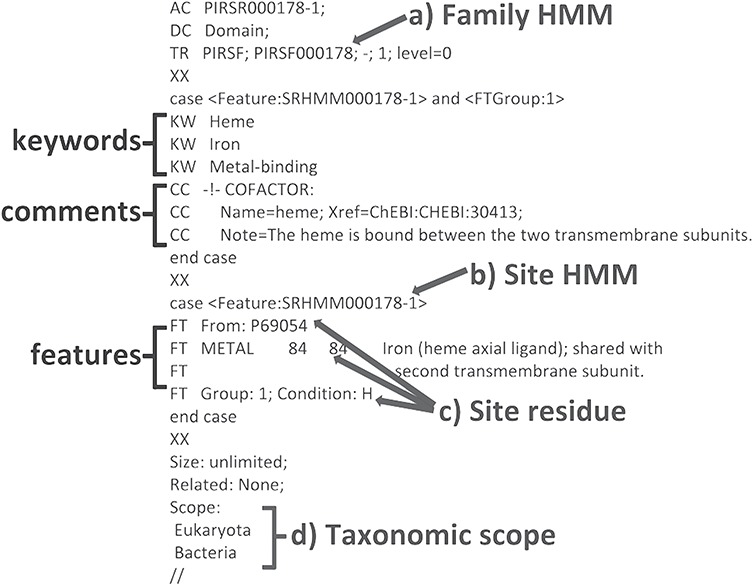
An example PIRSR (PIRSR000178-1) in UniRule flat file format. It specifies a set of test conditions that candidate uncharacterized proteins must pass to get corresponding annotations, including features with associated comments and keywords. The test conditions include the following: (**a**) a whole protein based family HMM (see TR); (**b**) a site-specific profile HMM (SRHMM); (**c**) functionally and structurally characterized residues of a manually curated template protein sequence; (**d**) the candidate protein is from an organism within the defined taxonomic scope.

We have developed a computational method that provides annotation of functional sites using position-specific conditional template annotation rules (PIRSRs; 21). Each rule specifies a set of match conditions that candidate proteins must pass in order to get the appropriate annotation of functionally important sites and regions. This process has generated high-quality annotations for UniProtKB/TrEMBL (automatically annotated and unreviewed; 24) protein sequences. PIRSRs are described in UniRule flat file format (.uru; ftp://ftp.expasy.org/databases/prosite/unirule.pdf). An example PIRSR is shown in Figure 2.

**Table 1:** Functional site feature types (https://web.expasy.org/docs/userman.html) supported by PIRSitePredict

**Feature types**	**Description**
ACT_SITE	Amino acid(s) involved in the activity of an enzyme
BINDING	Binding site for any chemical group (co-enzyme, prosthetic group, etc.)
CARBOHYD	Glycosylation site
CHAIN	Extent of a polypeptide chain in the mature protein
CROSSLNK	Post-translationally formed amino acid bonds
DISULFID	Disulfide bond
DNA_BIND	Extend of a DNA-binding region
LIPID	Covalent binding of a lipid moiety
METAL	Binding site for a metal ion
MOD_RES	Post-translational modification of a residue
MOTIF	Short (up to 20 amino acids) sequence motif of biological interest
NP_BIND	Extend of a nucleotide phosphate-binding region
PROPEP	Extent of a pro-peptide
REGION	Extent of a region of interest in the sequence
SITE	Any interesting single amino-acid site on the sequence, which is not defined by another feature key
ZN_FING	Extent of a zinc finger region

The overall PIRSR curation workflow is shown in the left box inside illustration in Figure 1. Internally, we have built a web-based user interface to facilitate the curation efforts. PIRSRs are defined starting with curated PIRSF/InterPro families that contain at least one known 3D structure with experimentally verified site information in published scientific literature. Characterized entries are selected as template proteins for PIRSR curation. For protein sequences where PIRSF assignment is unavailable but InterPro assignment is, PIRSR can be curated using InterPro signatures.

#### Build site-specific profile HMM

A set of UniProtKB/Swiss-Prot (24; annotated and reviewed by human experts) proteins in a given PIRSF/InterPro family including the template protein is used to create a multiple sequence alignment. Structure-guided manual editing of the alignment is done after visual inspection using an alignment editor to make sure that the residues of interest in the template are conserved among the aligned sequences. Conserved regions of the alignment covering the propagatable residues are concatenated to form the site-specific alignment. The reviewed (and in some cases edited) multiple sequence alignment is then used to build site-specific profile HMM model (SRHMM) using HMMER3 (25). The site-specific HMM is thus much more focused on the propagatable residues than the original full-length family HMM. The details can be found in (21).

#### Select site feature annotations

Various feature information about the candidate sites are derived from the annotations of chosen template protein, specifically, the annotation fields FT (Feature Table) (see feature types in Table 1 for details), with associated CC (comments) and KW (keywords) in UniProtKB/Swiss-Prot entries. Syntax and controlled vocabulary are used for site description and evidence attribution following UniProt curation standard.

#### Specify match condition

A set of match conditions is defined in the rule and must be met to enable prediction of annotations to a target protein sequence:

*Family HMM*: The target protein sequence must match the PIRSF/InterPro family HMM specified in the rule as ‘trigger’ condition [TR line].

*Taxonomic scope*: Rule can only be applied to a certain taxonomic branch, which is defined as Kingdom/sub-taxon in the ‘scope’ section [Scope block] in the rule.

*Site HMM*: Family HMM may not be suitable as a discriminator for a particular site of interest. The target protein must also match (with *e*-value threshold of 10^−4^) to the SRHMM defined as ‘feature group’ condition [Case statement] in the rule.

*Site residue*: The target and template protein sequences are aligned to the site-specific profile HMM. Target residues that match those defined as ‘feature table’ condition [FT lines] in the rule are eligible for prediction.

#### Prediction statistics

Each PIRSR is tested against all UniProtKB/Swiss-Prot members of the corresponding protein family by its performance statistics (Precision and Recall):

\begin{align*} Precision=&\ \frac{TP}{TP+ FP} \\ Recall=&\
\frac{TP}{TP+ FN} \end{align*}

where TP (True Positive), annotations that already exist in Swiss-Prot entries and are predicted by the rule; FP (False Positive), annotations that do not exist in the Swiss-Prot entries but are predicted by the rule; FN (False Negative), annotations that already exist in Swiss-Prot entries but is not predicted by the rule. The curators iteratively refine the rules based on the performance statistics.

### Implementation

PIRSitePredict is implemented in Java to ensure it can be used across different platforms. The software mainly consists of an IO (Input and Output) module and a prediction module. The IO module parses InterProScan XML file, PIRSR flat file, HMM file, FASTA file and GFF3 file. The IO module also generates the prediction results in different formats. The prediction module implements algorithms outlined in the right box inside illustration in Figure 1. PIRSitePredict is available as a downloadable stand-alone Java command line software package and also as an online prediction service, which was built on top of the stand-alone software package using Spring MVC 4, Thymeleaf, Bootstrap and jQuery.

PIRSitePredict can be run from the native operating system or in a Docker container. For online prediction service, a user can upload an InterProScan XML file, select a PIRSitePredict release (default, the latest release), specify the organism and HMMer *e*-value cutoff, then click Submit to start the prediction job. Each prediction job has a unique job ID and runs in the background. Once the job is finished and prediction results are ready, a link to the prediction results is presented to the user on the web page (and also via a notification email if the user has exercised that option). In addition to following the link to get the prediction results, the user can also use the job ID to retrieve the prediction results, which are stored for 30 days. The prediction results are presented as paginated tabular views. By using the search box at the top of the result table, the user can quickly filter the prediction results. Three buttons at the top of the table allow the filtered prediction results to be exported in TSV, XML or GFF3 formats. The PIRSR rule ID, Protein ID and Nucleotide ID columns are links to prediction results in rule-centric view, protein-centric view and nucleotide-centric view, respectively. A tutorial for using the command line tool and the online prediction service is available at https://research.bioinformatics.udel.edu/PIRSitePredict/documentation/standalone and https://research.bioinformatics.udel.edu/PIRSitePredict/documentation/online, respectively (see Supplementary file 1).

## Applications

### UniProtKB automatic annotation

**Figure 3:**
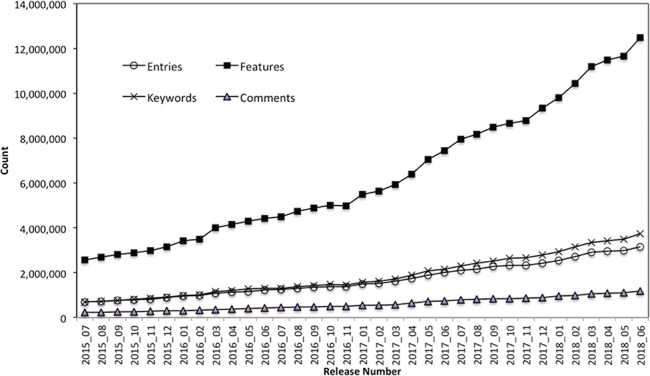
UniProtKB/TrEMBL protein sequence annotations generated by PIRSitePredict.

The PIRSitePredict software package has been integrated into UniProtKB automatic annotation production pipeline and provides high-quality annotations for UniProtKB/TrEMBL protein sequences on a monthly basis for 3 years. It takes protein sequences and other entry information from UniProtKB data files as input and generates the high-quality annotations for UniProtKB/TrEMBL sequences. Figure 3 shows the total number of annotations generated by PIRSitePredict over time.

As of release 2018_06, we have produced a total of 1006 PIRSRs that have provided annotations for 3 158 471 UniProt/TrEMBL entries. The average Precision and Recall over these PIRSRs are 91% and 85%, respectively. For those rules with lower precision and recall, they are further reviewed and refined by the curators. Overall, PIRSitePredict supports 16 functional site annotation types (https://web.expasy.org/docs/userman.html) as shown in Table 1. These functional site features (FT) are collected from UniProtKB/Swiss-Prot template protein sequence annotations. We also collect other related annotations, such as keywords (KW) and comments (CC), and specify them in the PIRSRs.

### Genome/Transcriptome annotation

**Figure 4:**
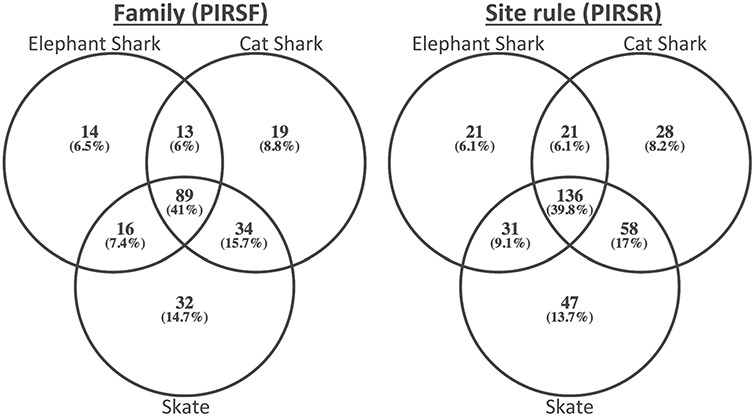
The Venn diagrams of overlapping families (left) and rules (right) for embryonic transcriptomes of three cartilaginous fishes.

To demonstrate its usefulness to the genomics community, we used PIRSitePredict to annotate uncharacterized proteins from Trinity (26) RNA-seq *de novo* assembly of embryonic transcriptomes of the following three cartilaginous fishes (27): *Leucoraja erinacea* (Little Skate), *Scyliorhinus canicula* (Small-spotted Catshark) and *Callorhinchus milii* (Elephant Shark). The summary of predicted annotations is shown in Table 2. On average about 1200 lines of annotations were predicted for each species. Figure 4 shows the Venn diagrams of overlapping families/rules.

**Table 2:** Summary of predicted annotations for embryonic transcriptomes of three cartilaginous fishes

	**Little Skate**	**Small-spotted Catshark**	**Elephant Shark**
Transcriptome Contigs	103 996	107 231	92 334
PIRSRs Applicable	272	243	209
Proteins Annotated	251	241	191
Annotations Predicted	1342	1259	991
Features (FT)	1021	955	728
Keywords (KW)	255	246	210
Comments (CC)	66	58	53

We ran InterProScan (version: 5.25–64.0) against three transcriptome assembly contigs in FASTA format to get three InterProScan XML output files. We then applied the PIRSitePredict package (2018_06) to those XML files to evaluate the performance of our software. The evaluation was performed on Fedora Core 25 x86_64 Linux server with 256G RAM and 48 Intel(R) Xeon(R) CPU E5-2687W v4 @ 3.00GHz. For each InterProScan XML file, we ran the software 10 times to get the average memory usage and average runtime. The performance evaluation results are shown in Table 3. It is clear that PIRSitePredict runs very fast and has a very small memory footprint.

**Table 3:** Performance evaluation of PIRSitePredict software

**Little Skate**		**Small-spotted Catshark**		**Elephant Shark**	
Memory usage (Mbytes)	Runtime (m:ss)	Memory usage (Mbytes)	Runtime (m:ss)	Memory usage (Mbytes)	Runtime (m:ss)
983	01:38.2	995	01:33.6	679	01:15.7

We also compared the annotations of three cartilaginous fishes predicted by PIRSitePredict with those predicted by High-quality Automated and Manual Annotation of Proteins (HAMAP; 28). HAMAP provides manually curated profiles for protein sequence family classification and expert-curated rules for functional annotation of family members. Like PIRSitePredict, HAMAP supports annotation of functionally important sites (such as ion-, substrate- and cofactor-binding sites, catalytic residues and post-translational modifications), and protein sequences can be classified and annotated through the HAMAP-Scan (https://hamap.expasy.org/hamap_scan.html) web site.

We used the HAMAP-Scan to analyze the protein sequences from the three cartilaginous fishes’ transcriptomes for which PIRSitePredict predicated the annotations, then compared the annotation results from the two tools. In general, for those proteins annotated by PIRSitePredict, <5% of them were annotated by HAMAP (due predominantly to the differences in family membership). However, for those proteins where membership overlaps in each system, and for annotations predicted by both PIRSitePredict and HAMAP, >90% are the same. Overall, HAMAP rules provide other annotation types in addition to site-related (e.g. protein names, gene names, function, catalytic activity and Gene Ontology terms). In contrast, PIRSRs only focus on predicting functional site-related annotations. The detailed comparison results are described in an additional data file (see Supplementary file 2).

**Figure 5:**
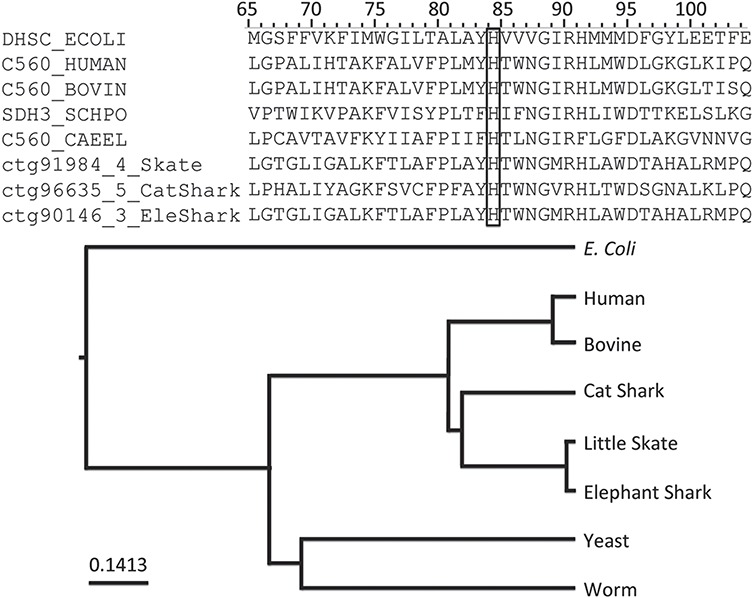
An application of functional site prediction with PIRSitePredict using PIRSR000178-1 as an example. The template sequence for the site rule PIRSR000178-1 (see Figure 2) is P69054 (UniProtKB Accession), which is *E. coli* SDH cytochrome b556 subunit. The multiple sequence alignment and phylogenetic tree for eight protein sequences matching the conditions of PIRSR000178-1 were generated with Seqotron (29). The sequences are for corresponding proteins from *E. coli*, human, bovine, yeast, worm, little skate, small-spotted catshark and elephant shark, respectively. The conserved metal-binding site histidine is marked with a box, and the numbers on the top correspond to the template sequence P69054 (*E. coli*).

Among the annotations predicted (Table 2), we found that rule PIRSR000178-1 (see Figure 2) is applicable to all three cartilaginous fish embryonic transcriptomes and to the human mitochondrial proteome. PIRSR000178-1 defines a metal-binding site important for heme binding in succinate dehydrogenase (SDH) cytochrome subunits. Figure 5 shows the multiple sequence alignment and phylogenetic tree for the sequences from three cartilaginous fishes, human, bovine, worm, yeast and *Escherichia coli* that satisfy the PIRSR000178-1’s conditions. The heme iron-binding histidine site is conserved in all eight sequences. As expected from phylogeny, the sequences from three cartilaginous fishes clustered as a group, with these being more similar to human and bovine sequences than to those of yeast, worm and *E. coli*. Altogether, the results provide not only annotation for the heme iron-binding sites with relevant keywords and comments, but also provide indication that functional SDH is present in these fishes.

## Discussion

In PIRSR, a set of position-specific conditional template annotations is curated from template protein and specified as rule to indicate the conditions whereby candidates for annotation must pass. Briefly, these are the following: (i) if the protein belongs to a family that contains proteins related to one with the supposed activity; (ii) if the protein contains the conserved regions found in proteins known to have the supposed activity; and (iii) if the protein contains the precise amino acids required for the supposed activity. In contrast to other types of prediction, for example, family-based prediction, rule-based approach increases the specificity by combining information from sequence, structure, domains, motifs and common ancestry to both make predictions of global function and to provide annotation (herein called ‘features’) to individual amino acids.

In this paper, we demonstrate the ability of PIRSitePredict to serve as a module in the functional annotation of a *de novo* transcriptome assembly project. PIRSitePredict can also be used to reveal similarities and differences in transcriptomes by focusing on sequences with PIRSR annotations. For example, potential orthologs (with functional sites predicted) for a subset of human mitochondrial proteins (see Genome/Transcriptome annotation section) in the embryonic transcriptome of Little Skate, Small-spotted Catshark and Elephant Shark were efficiently identified using results generated by PIRSitePredict.

Currently, target protein sequences must be processed by InterProScan before being annotated by PIRSitePredict because one of the match conditions in PIRSRs is that the target protein sequence must match the PIRSF/InterPro family HMM specified in the rule. Additional study is needed to see if we can remove this restriction and still get confident high-quality annotations. If so, our tool will be able to do prediction using protein sequences in FASTA format directly instead of InterProScan XML format.

Both HAMAP and PIRSitePredict have been successfully implemented to annotate UniProtKB/TrEMBL protein sequences in UniRule for a number of years. However, PIRSitePredict is now available as a downloadable stand-alone Java command line software package for use by those seeking to add site-specific functional annotation to their annotation pipelines.

## Conclusion

Fine-grained ‘local’ annotation of functional sites at the level of individual amino acid can be achieved with PIRSitePredict. It enables streamlined functional site annotation of protein sequences and can be used in the downstream functional annotation of *de novo* genome/transcriptome assembly project. A downloadable stand-alone Java command line software package and an online prediction service are available at the PIRSitePredict website.

## Supplementary Material

Supplementary Data

## Notes

Database URL: https://research.bioinformatics.udel.edu/PIRSitePredict/
