# Supplementary material for: PIRSitePredict for protein functional site prediction using position-specific rules
Source: Database (Oxford). 2019 Feb 26;2019:baz026. doi: 10.1093/database/baz026 (PMC6389862; doi:10.1093/database/baz026)

**Tutorial - Online Prediction from PIRSitePredict Website**

https://research.bioinformatics.udel.edu/PIRSitePredict/prediction/service

Table of Content

- Online submission form
- View your job status
- Retrieve the production results
- View the prediction results
  - Tabular view
  - Rule centric view
  - Rule HTML view
  - Protein centric view
  - Nucleotide centric view

Online submission form

To use online prediction service, user can upload an InterProScan XML file, select PIRSitePredict release they want to use (default: the latest release), specify the organism and HMMer e-value cutoff. Optionally, user can enter his/her email to be notified once the prediction job is finished and the results are ready. Click **Submit** to start the prediction job.


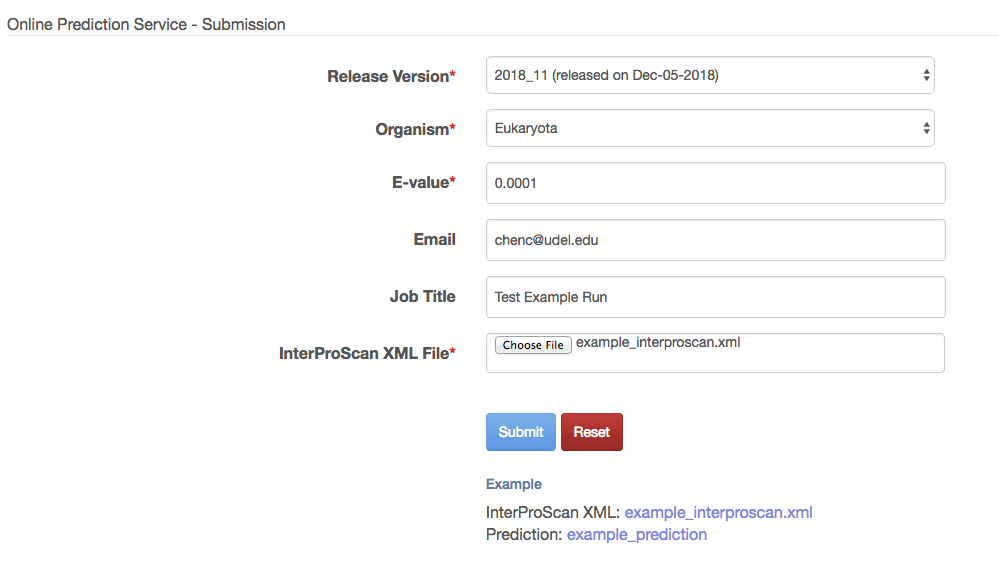


View your job status

Each prediction job has a unique job ID, and will be run in the background. However, the detailed job status will be presented to the user. Once the job is finished and the prediction results are ready, a link to the prediction results is presented to the user. The same link will be presented in the notification email if user has provided his/her email when the job was submitted.


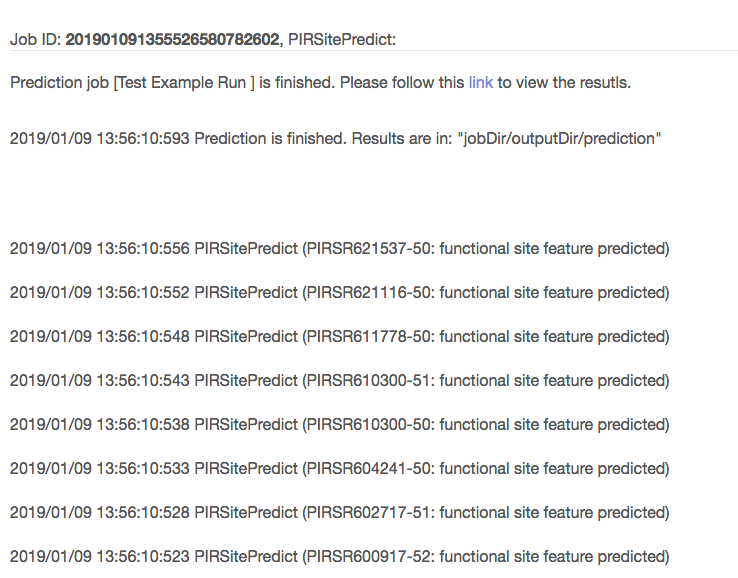


Retrieval the prediction results

In addition to the link presented to the user to view the results, user can enter the job ID to retrieve the prediction results. To do this, select “Retrieve prediction results” under the prediction service menu option.


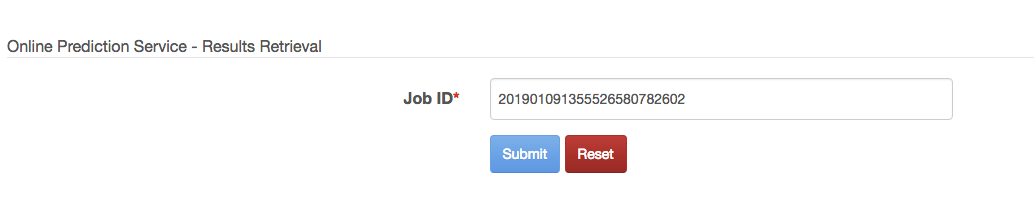


Prediction results - tabular view

The prediction results are presented as paginated tabular views. By using the search box on top of the table, user can quickly filter the predictions. Three buttons on the top-right corner of the table allow user the export the (filtered) prediction results in tab-separated values (TSV), Extensible Markup Language (XML) and Generic Feature Format (GFF3) formats.


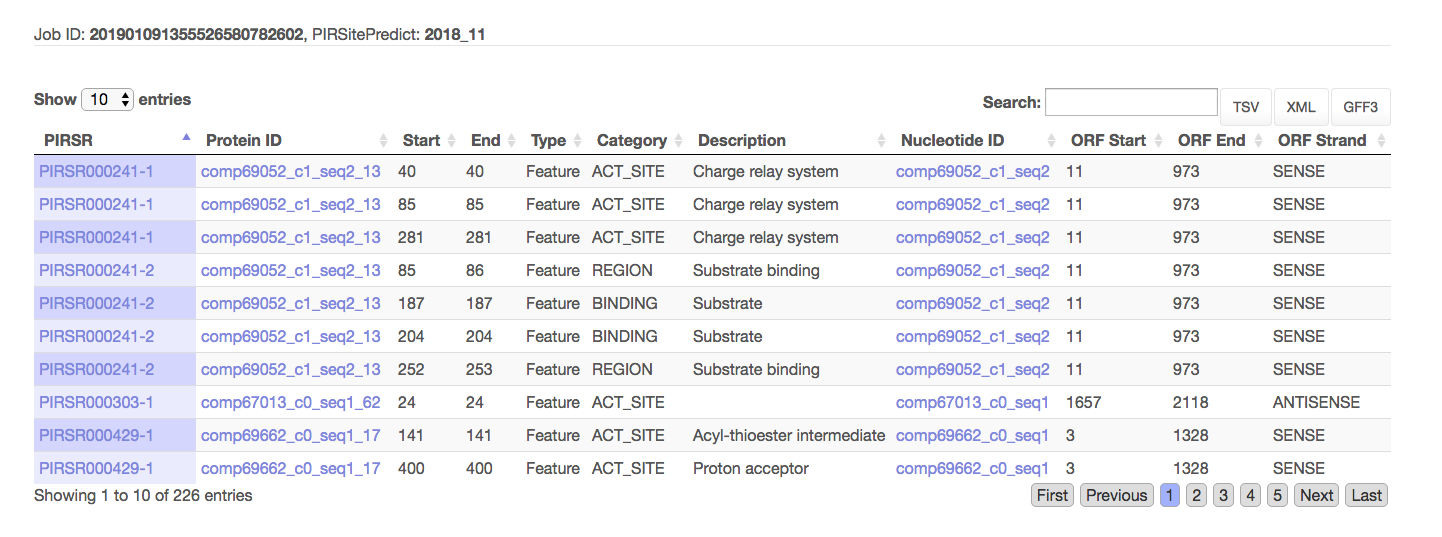


The IDs in The PIRSR rule ID, Protein ID, and Nucleotide ID columns are links to prediction results in rule centric view, protein centric view and nucleotide centric view, respectively. For details see sections below.

Prediction results - rule centric view

Rule centric view presents the results predicted by the given rule in the given prediction job. The left column lists the proteins that have functional sites prediction by the given rule. By clicking each protein ID on the left, the right column will show the detailed prediction results (Features, Comments, Keywords) as well as the protein amino acid sequences. By clicking the icon next to each prediction result, a pop-up box will appear with source information (link to PIRSR rule that generates this prediction).


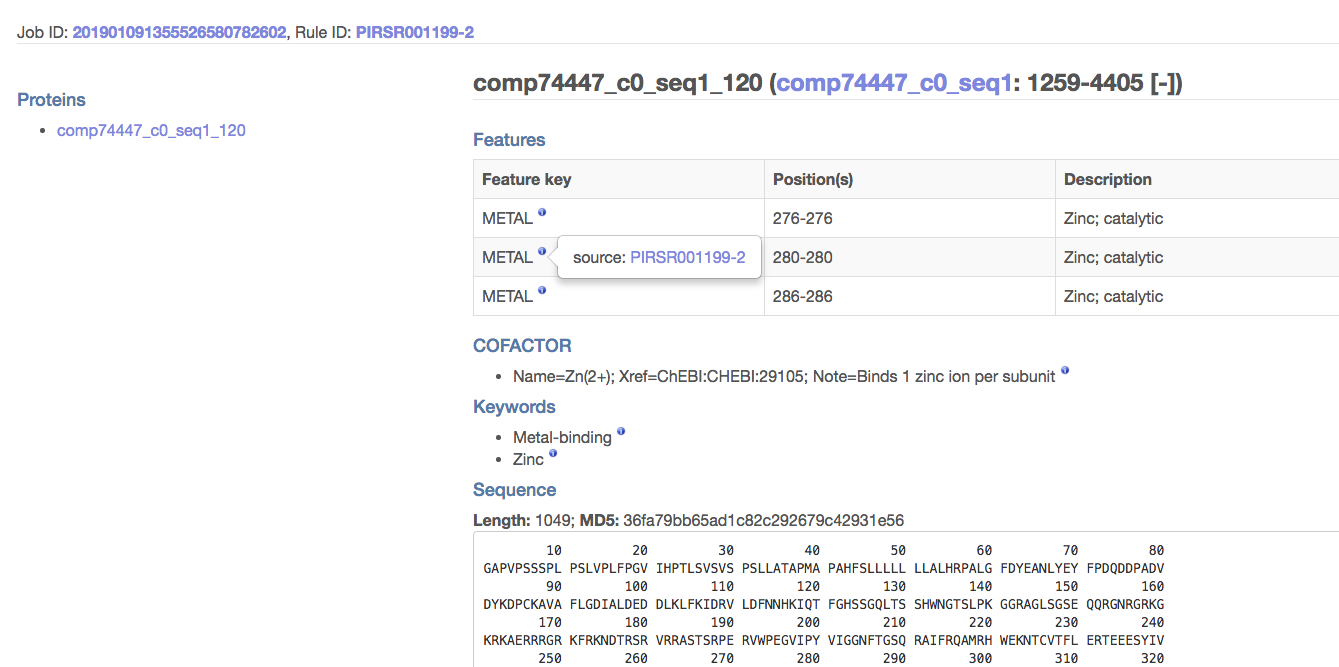


Prediction results - rule HTML view

By clicking the PIRSR rule ID link from the rule centric view, a HTML view of PIRSR rule will be presented.


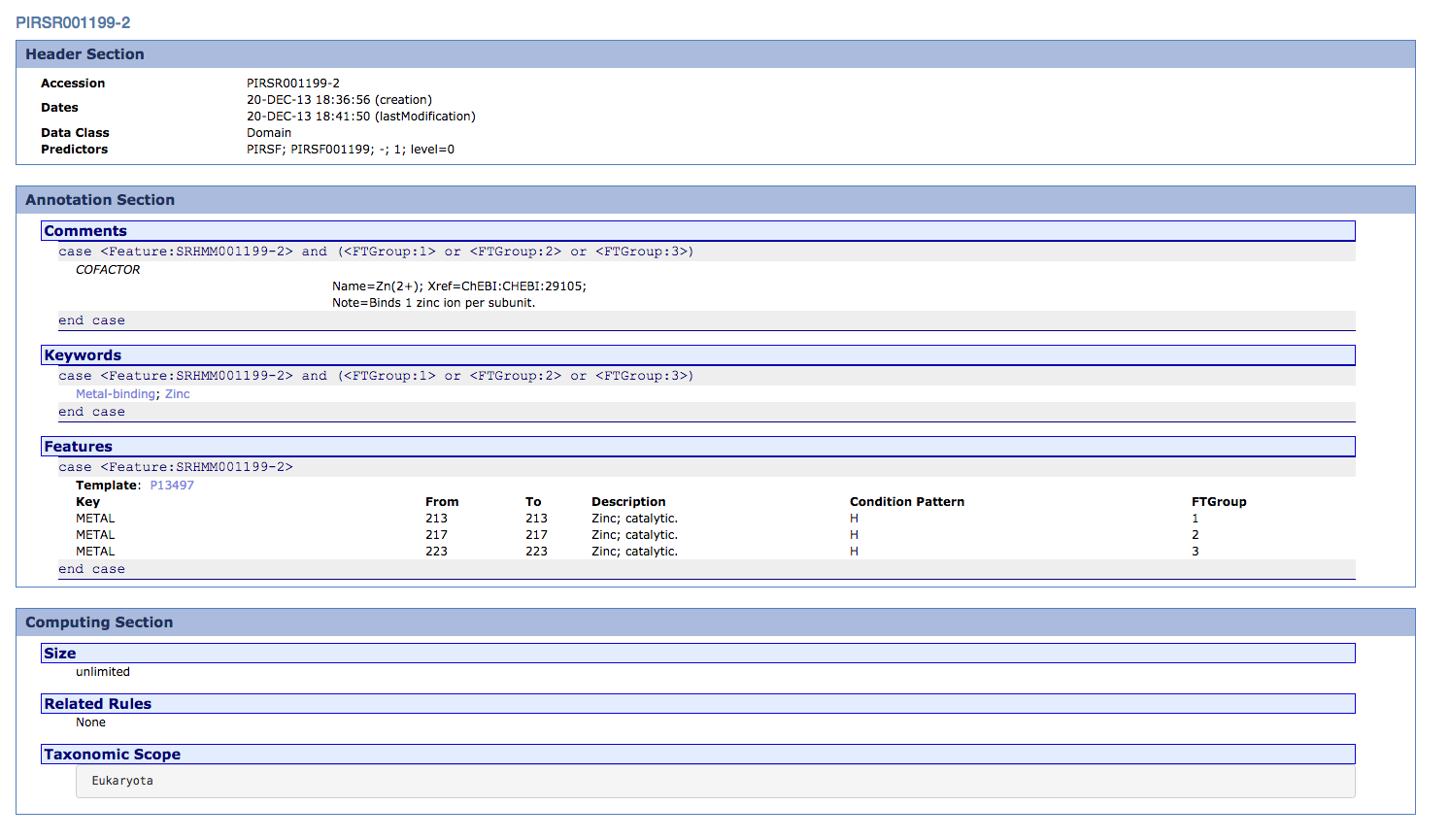

Prediction results - protein centric view

Protein centric view presents the results predicted by all the relevant PIRSR rules in the given prediction job with detailed prediction results (Features, Comments, Keywords) as well as the protein amino acids sequences. By clicking the icon next to each prediction result, a pop-up box will appear with source information (link to PIRSR rule that generates this prediction).


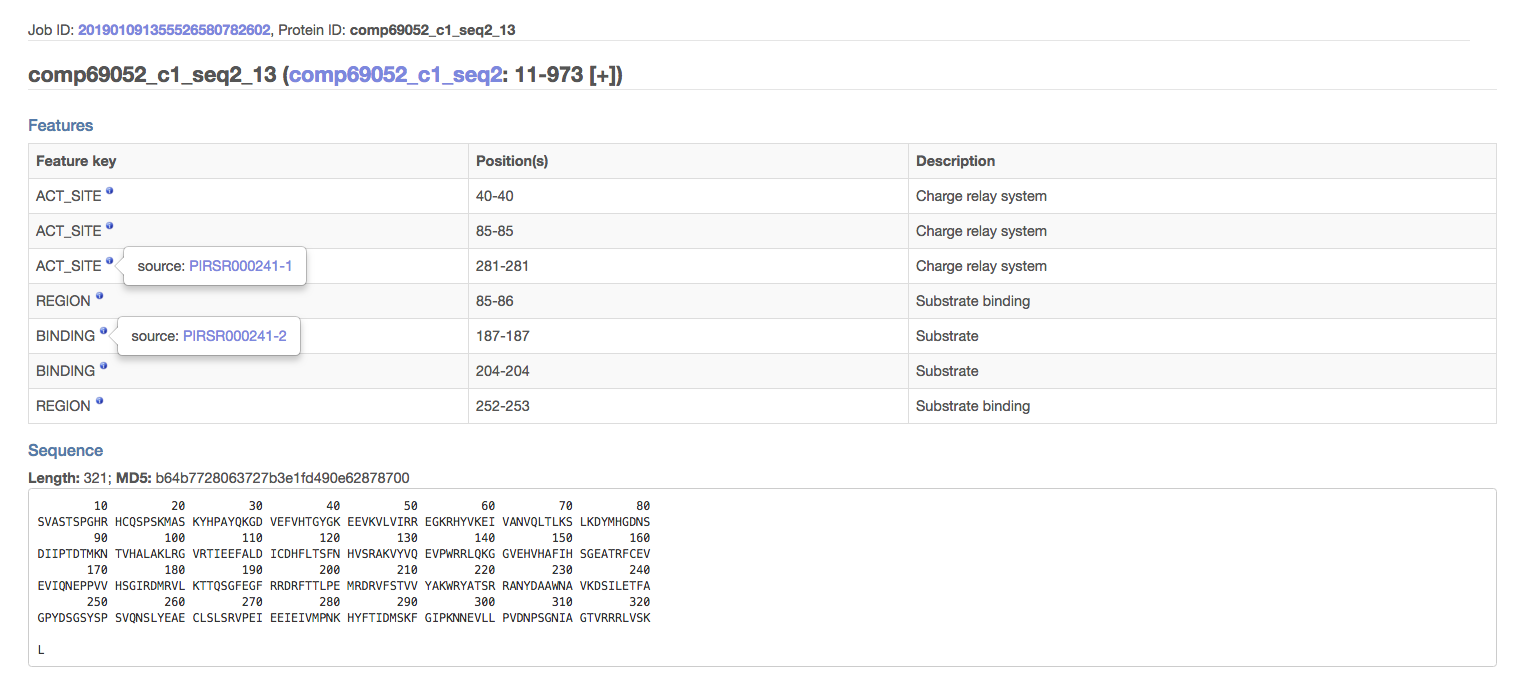


Prediction results - nucleotide centric view

Nucleotide centric view presents the results for proteins derived from the ORF of the given nucleotide sequence in the given prediction job. The left column lists the proteins that are derived from the ORF of the given nucleotide sequence and have functional sites prediction by the PIRSR rules. The right column starts with the nucleotide sequence, followed by the detailed prediction results (Features, Comments, Keywords) as well as the protein amino acids sequences of the proteins listed on the left column.


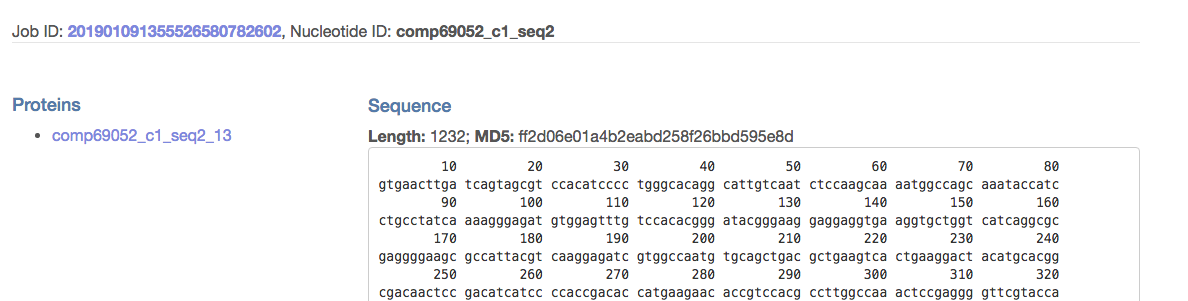


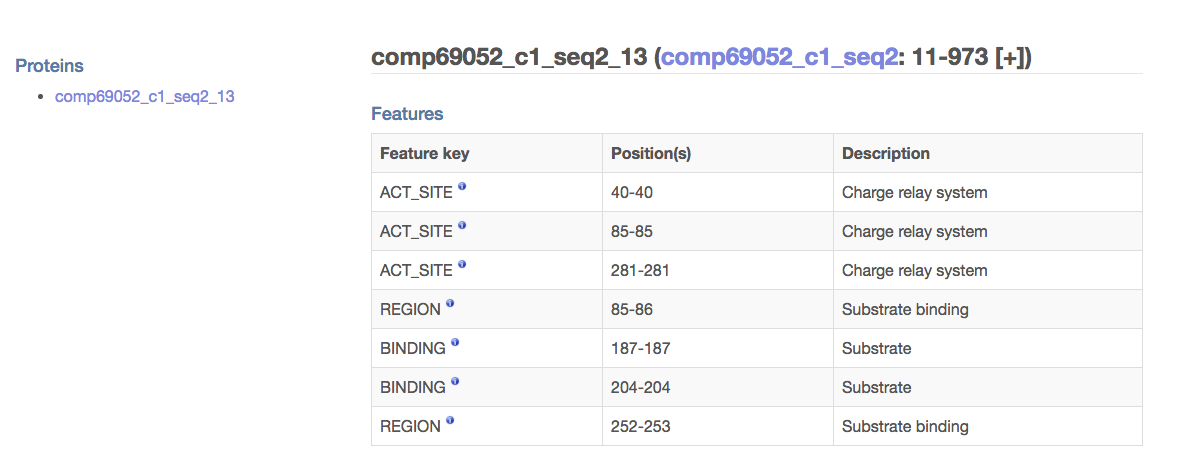


**Tutorial - Running PIRSitePredict from command line**

### Download the stand alone software package from our website: <https://research.bioinformatics.udel.edu/PIRSitePredict/download>

### You can run PIRSitePredict either from the native OS or from a dock container:

### From Native OS

#### Requirement:

- Java 8. Please follow this [link](http://www.oracle.com/technetwork/java/javase/downloads/index.html) to download and install.
- Latest HMMER software. Please follow this [link](http://hmmer.janelia.org/download.html) to download and install.

One you have uncompressed your download, you can run it directly from the command line.

Run the supplied shell script. If you run this script with no arguments, you will be presented with the usage instructions:


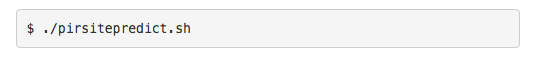


You will see the following **usage instructions**:


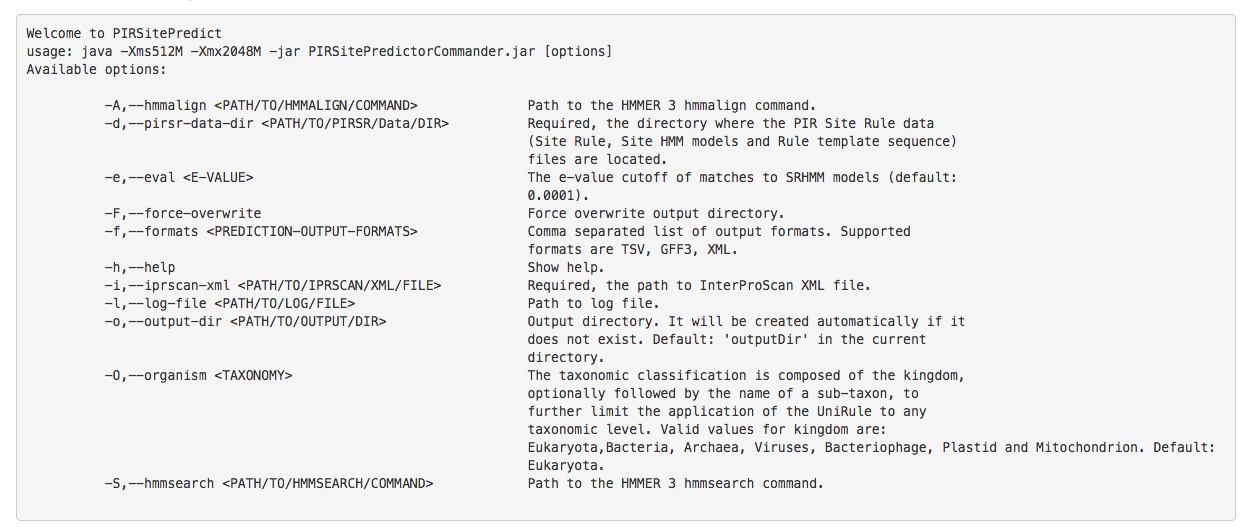


PIRSitePredict test run

The distribution of PIRSitePredict provides a InterProScan XML test file ([example/example_interproscan.xml](https://research.bioinformatics.udel.edu/PIRSitePredict/download/example_interproscan)) and its corresponding predictions in TSV, XML and GFF4 formats:

- [example/example_interproscan_prediction.tsv](https://research.bioinformatics.udel.edu/PIRSitePredict/download/example_prediction/tsv)
- [example/example_interproscan_prediction.xml](https://research.bioinformatics.udel.edu/PIRSitePredict/download/example_prediction/xml)
- [example/example_interproscan_prediction.gff3](https://research.bioinformatics.udel.edu/PIRSitePredict/download/example_prediction/gff3)

which you can use to check how PIRSitePredict behaves on your system. Run the following command:


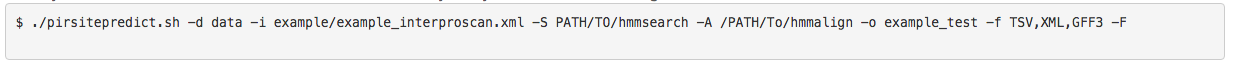


The standard output will be something like below:


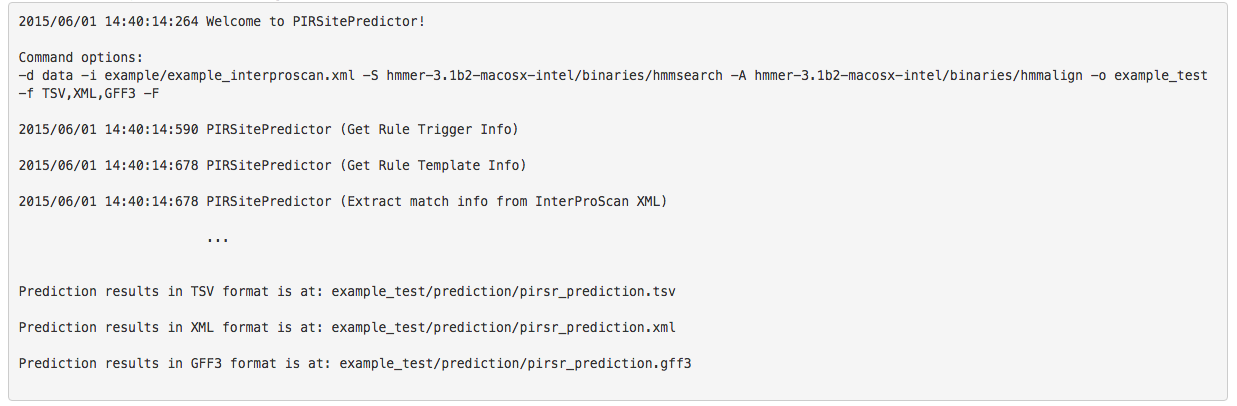


### From Docker Container

Set up local working directory to hold input and output files. It will be mounted into Docker container.


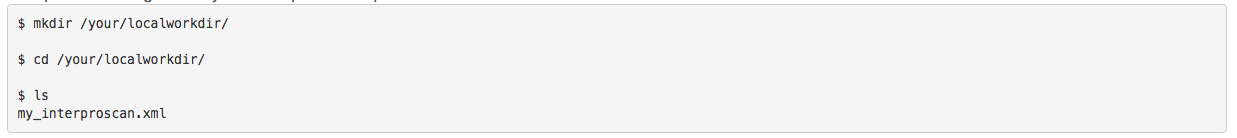


Test PIRSitePredict run using Docker:


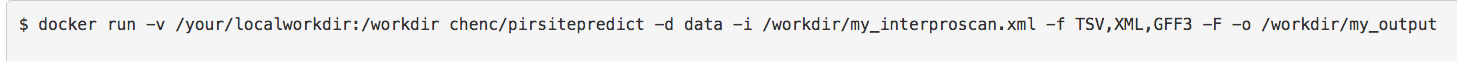

Supplement: Supplementary Data [file baz026_supp.zip › Additional file 1.docx]
